# Study on the Influence of Ultrafast Laser Welding Parameters on Glass Bonding Performance

**DOI:** 10.3390/mi16080888

**Published:** 2025-07-30

**Authors:** Aowei Xing, Ziwei Li, Tianfeng Zhou, Zhiyuan Huang, Weijia Guo, Peng Liu

**Affiliations:** 1 School of Mechanical Engineering, Beijing Institute of Technology, Beijing 100081, China; xaw5960@163.com (A.X.); 3220240390@bit.edu.cn (Z.L.); zhoutf@bit.edu.cn (T.Z.); 15729638710@163.com (Z.H.); guo_weijia@bit.edu.cn (W.G.); 2 State Key Laboratory of Chips and Systems for Advanced Light Field Display, School of Mechanical Engineering, Beijing Institute of Technology, Beijing 100081, China; 3 Chongqing Innovation Center, Beijing Institute of Technology, Chongqing 401120, China

**Keywords:** glass bonding, laser bonding, microfluidic chip, weld morphologies

## Abstract

Glass enjoys a wide range of applications thanks to its superior optical properties and chemical stability. Conventional glass bonding techniques suffer from low efficiency, limited precision, and high cost. Moreover, for multilayer glass bonding, repeated alignment is often required, further complicating the process. These limitations have become major constraints on the advancement of microfluidic chip technologies. Laser bonding of microfluidic chips offers high precision and efficiency. This research first uses an ultrafast laser system to investigate how processing parameters affect weld morphology, identifying the optimal parameter range. Then, this paper proposes two methods for ultrafast-laser bonding of multilayer glass with different thicknesses and performs preliminary experiments to demonstrate their feasibility. The research in this paper could expand the fabrication method of microfluidic chips and lay a foundation for the wider application of microfluidic chips.

## 1. Introduction

Glass is widely utilized in optics, biology, and medicine due to its superior optical properties and chemical stability. A microfluidic chip integrates sample preparation, reaction, and detection processes for biological, chemical, and medical assays on a single device. Its core components are micro-reactors and flow channels, through which fluids and droplets move to carry out automated reactions. Hence, it is also called a “lab-on-a-chip”. Microfluidic chips have considerable potential in the life sciences, chemistry and medicine [1]. It has emerged as a cutting-edge field that integrates multiple disciplines such as mechanics, electronics, materials, biology, chemistry and medicine.

Bonding refers to the tight joining of two substrates—identical or dissimilar—via chemical bonds or intermolecular forces following surface cleaning and activation. In the fabrication process of microfluidic chips, it is usually necessary to seal the microminiature flow channel into the glass interior; therefore, the bonding method for glass has become a key factor limiting microfluidic-chip performance [2]. The common methods of glass bonding today are thermal bonding [3], low-temperature bonding [4], anode bonding [5] and adhesive bonding [6], etc.

Hot (direct) bonding typically proceeds as follows: two glass substrates are cleaned and brought into intimate contact in a dust-free environment. They are then sandwiched between high-temperature-resistant plates and subjected to vacuum heating and pressure. The bonding success strongly depends on surface flatness, roughness, and cleanliness. This method requires long processing times and specialized equipment.

Low-temperature bonding refers to the bonding of glass without the use of a high-temperature environment, and generally refers to surface-activated bonding and interlayer bonding. Surface-activated bonding includes chemical activation bonding [7] and plasma-activated bonding [8]. Chemical activation bonding refers to the use of corrosive chemical reagents such as concentrated sulfuric acid or hydrofluoric acid to treat the glass, so as to form a hydrated film on its surface, and then low-temperature heating to convert the Si-ONa inside the film into Si-OH, and finally, the Si-OH on the two pieces of glass is dehydrated and condensed to form a strong Si-O-Si bond. Chemical activation bonding requires the use of hazardous chemical reagents, which reduces the safety of the experiment and is prone to safety hazards. Plasma-activated bonding is a similar process, in which oxygen plasma is bombarded on the surface to increase the density of suspended bonds on the surface, which then react quickly to form natural atomic bonds upon contact. This method does not require the use of hazardous reagents, thus improving the safety of the experiment, but the plasma activation equipment is expensive, which will increase the cost of the experiment. Interlayer bonding refers to the pre-spraying of a low-melting-point, high-mobility medium layer on the surface of the two glasses to be bonded, and then annealing them at low temperatures to realize the bonding between the glasses.

Anodic bonding, also known as electrostatic bonding and field-assisted bonding, requires the thermal dissociation of basic oxides within the glass by elevated temperatures. At high temperatures, the ionic conductivity of the glass increases. Taking advantage of this ionic conductivity, an electric field is applied to move the positive alkaline ions toward the cathode and away from the bonding surfaces. The contact point is polarized due to the electric field generated when the bonding surfaces are close together. Positive ions diffusing from the bonding interface generate free oxygen ions, which chemically bond with silicon, forming Si-O-Si covalent bonds [5]. This method is not suitable for bonding materials with different thermal expansion rates because of the heating required; otherwise, large stresses will be generated during the cooling process, leading to breakage after bonding, etc.

Adhesive bonding refers to the use of the spin-coating method of UV adhesive evenly coated on the bonding surface, and then the two bonding surfaces are brought into contact with the UV light irradiation, curing can be completed, or the use of heating curing adhesive for heating bonding. This method has a wide range of applications because of its convenience and simplicity of operation, but its strength and stability in chemical reactions limit its application in some scenarios.

At present, thermal bonding, low-temperature bonding, anodic bonding, adhesive bonding and other bonding methods in terms of bond strength, stability, processing efficiency and sealing, etc. have their own strengths and weaknesses and scope of application, and can not meet a variety of requirements at the same time.

Laser technology, as an emerging technique, has found widespread applications in various fields, such as cutting, surface pretreatment, and tribology. Shin et al. [9] investigated the femtosecond laser ablation process for cutting 100 μm thick thin-aluminum borosilicate glass, focusing on the effective cutting speed and mechanical strength of the cut samples. Three-point bending tests were conducted to evaluate the bending strength of the samples. Sorrentino et al. [10] explored an innovative CO_2_ pulsed laser pretreatment method for bonding carbon fiber-reinforced composites. They fabricated notched bending samples using various laser treatment densities and obtained corresponding fracture toughness values using the flexure-based beam method. Additionally, lasers have also gained attention in surface texturing due to their high flexibility, excellent texturing precision, and good controllability. Mao et al. [11] discussed the process design of laser shock texturing (LST) using direct laser ablation, laser interference, and laser shock processing, with a focus on the effects of laser parameters on the texturing characteristics.

Laser welding refers to the use of laser penetration, so that the laser can penetrate the upper layer of glass, focus on the surface to be bonded, and use the laser energy to complete the welding [12]. Lasers can be categorized into long-pulse lasers and ultrafast lasers according to their pulse widths. In the use of long-pulse laser welding, due to the transmittance of the glass material, the laser wavelength that can be absorbed by the glass will be absorbed on the surface of the glass but not on the bonding surface, and the laser wavelength that can pass through the glass will pass through the glass directly, so it is generally necessary to add an intermediate absorbing material between the glass with opaque material, which affects the transmittance of the glass [13]. Ultra-fast laser is also called ultrashort pulse laser, it refers to the pulse width in picoseconds below the laser, in the use of ultrafast laser welding, because of its pulse time scale is very short, can avoid the long pulse laser produced by the melting of the material and the phenomenon of continuous evaporation, greatly improving the quality of the process, and at the same time, it can be with transparent materials and the occurrence of nonlinear absorption effect and the deposition of energy [14]. This makes it possible to weld glass without the use of an intermediate absorber layer or an opaque material underneath, greatly improving the application scenarios and scope of laser welding.

In 2005, Tamaki et al. [15], from Osaka University in Japan, first proposed and experimentally demonstrated the application of low repetition rate femtosecond pulse laser in selective welding of fused silica glass, which opened the prelude to the research of ultrafast laser direct selective welding. Subsequently, Tamaki et al. [16] from Osaka University in Japan introduced high repetition rate femtosecond laser pulses and realized direct selective welding between different types of glass materials and selective welding between glass and single crystal silicon semiconductor materials through the laser-induced thermal accumulation effect. In 2011, Sugioka et al. [17], from RIKEN, proposed the mechanism of double pulse action. By precisely controlling the time interval of double pulses, the team successfully greatly improved the laser energy deposition efficiency at low repetition rate, thereby increasing the laser welding strength by 22%.

Compared with low repetition rate ultrafast laser, high repetition rate ultrafast laser welding can have higher energy deposition efficiency and higher welding speed [18,19]. Therefore, the study of the action characteristics of high repetition rate ultrafast lasers has become a major focus of ultrafast laser selective welding.

In 2011, Isamu Miyamoto et al. [20] from Osaka University in Japan and the Graduate School of Advanced Optical Technology at the University of Erlangen-Nuremberg in Germany constructed a heat conduction model and used it to simulate the nonlinear absorption characteristics of ultrashort laser pulses in the process of modifying the interior of large pieces of glass. The study found that with the increase of laser pulse energy and repetition frequency, the nonlinear absorbance will also increase accordingly, and can even reach 90% at the highest. At the same time, with the increase of the average laser absorption power, the laser absorption area will expand toward the laser source. Based on this, the use of high repetition rate ultrafast laser burst mode for selective material welding can not only ensure high energy absorption efficiency, but also effectively control the laser-induced heat accumulation effect and optimize the welding quality [16]. In 2016, Sören Richter et al. [21], from the Institute of Applied Physics of the Abbe Center for Photonics in Germany, conducted welding experiments on fused quartz, borosilicate glass and glass with a low thermal expansion coefficient, and determined that laser-induced stress is the limiting factor for the stability of welding glass.

With the continuous advancement of research work, ultrafast laser welding has also been successfully applied to transparent crystals and metals, glass, high melting point semiconductors, transparent ceramics, and organic polymers, showing extremely high material applicability. In 2019, Penilla et al. [22], from the University of California, USA, used ultrafast lasers to encapsulate transparent ceramics and electronic components. In 2022, Yu Long et al. [23] from Guangxi University used femtosecond lasers to perform heterogeneous material bonding of glass and Kovar metal under non-optical contact conditions. Glass-copper, glass-al6063 and sapphire glass-ceramics were also welded together. This work greatly simplified the femtosecond laser micro-welding process and promoted its industrial application in optoelectronic devices, medical devices, MEMS and other fields.

Although laser bonding offers unique advantages such as high precision and efficiency, the technology is still relatively new, and many of its potential application scenarios remain to be explored by researchers. In this paper, by using the laser experimental system, we investigated the influence of ultrafast laser processing parameters on weld morphologies to find the relatively optimal laser processing parameters; secondly, we proposed two methods of ultrafast laser bonding of multilayer glass with different thicknesses, and made exploratory experiments on them. This study will promote the development of laser bonding technology for microfluidic chips and bring broad prospects for the diversified design and precision manufacturing of microfluidic chips.

## 2. Experimental Materials and Methods

### 2.1. Laboratory Equipment

**Figure 1:**
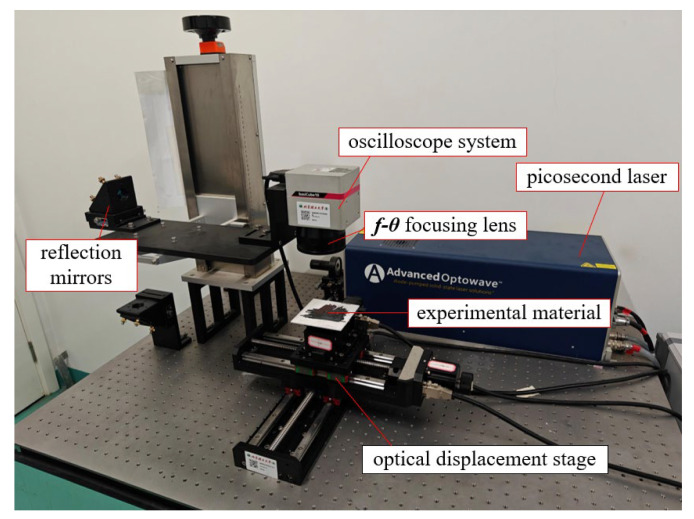
Ultra-fast laser etching processing system.

The ultrafast laser etching and processing system consists of a picosecond laser, an optical displacement stage, an 8× beam expander, a water cooling system, three total reflection mirrors, a 100 mm focal length *f*-θ focusing lens and the ScanLab oscilloscope system, which is illustrated in Figure 1. The scanner system is BasiCube 10-1064 nm, and the table and scanner system are controlled by Samlight software for precision motion control up to 10 μm, which can also plan the laser scanning path. The picosecond laser is an Advanced Optowave diode-pumped solid-state laser (AMT-1064-20-100-W); its output power and repetition rate are software-controlled. Detailed parameters of the processing system are given in Table 1.

### 2.2. Laboratory Materials

We used soda-lime glass (SiO_2_, CaO, and Na_2_O) as the test substrate. It is the most commonly used glass material for microfluidic chips, and also the most common glass in daily life. It has stable chemical properties, do not react with most of the chemical reagents, which is conducive to the microfluidic chip reaction; has good optical transmittance, which is conducive to the observation and detection of the reaction, has good mechanical strength, can be a certain degree of mechanical processing to meet the shape of the requirements; has good thermal stability, can withstand a certain degree of temperature change. In addition, the use of soda-lime glass for experiments also helps to reduce the cost of experiments. The physical properties of soda-lime glass are shown in Table 2.

Prior to the experiment, the soda-lime glass was cut into small pieces (20 mm × 50 mm × 1.5 mm), ultrasonically cleaned with anhydrous ethanol for 5 min, followed by deionized water for another 5 min, and then dried for use.

### 2.3. Experimental Methods

After the focusing treatment using the focusing lens and ITO glass, the two pre-treated glass were tightly fitted until the appearance of uniform interference fringes, and no fixture was used in the experimental process in order to avoid glass breakage due to the introduction of large stresses during the laser bonding process, after which the glass was placed on the bench, and the focal point was firstly moved to the upper surface of the glass, and then shifted downward by 0.93 mm, at which time, the laser The focal point in the role of glass refraction, the distance moved in the glass is

(1) $$\Delta z^{\prime }=n\Delta z$$

In the equation, Δz=0.93,n=1.52 resulted in $\Delta z^{\prime }=1.4136$. In this case, the distance of the laser focus from the surface to be bonded is 86 μm (The distance between the naturally stacked glass surfaces is about 3 μm. In this experiment, we do not consider its effect for the time being), and carry out the scribing experiment on the plane perpendicular to the laser propagation direction to observe the effect of laser parameters on the weld morphologies, and finally choose the relatively better parameters.

In this experiment, there are four process parameters affecting the weld morphologies after bonding, which are single pulse energy, scanning speed, repetition frequency and scanning times. Among them, the single pulse energy is controlled by the laser output frequency, and the output power is controlled by the change of the power code in the laser; the output power is measured by the power meter, and the number of repetitions of each group of experimental parameters is five times.

At present, there are no explicit international technical standards or guidelines for evaluating the quality of laser bonding. However, assessment is generally based on the morphology of the bonded area and the bonding strength. A high-quality weld should be as flat and uniform as possible, free of cracks, voids, bubbles, or other defects. The weld width should be moderate, and the heat-affected zone should be kept as narrow as possible. Since higher bonding strength is desirable, optimal process parameters should be determined by balancing weld morphology and bonding strength.

The weld morphologies and the heat-affected zone were characterized using a Keyence laser confocal scanning microscope (VK-X260K). A 50× objective lens was employed to observe the bonding interface in detail and to measure the weld width and heat-affected zone width. The high-resolution images obtained from the confocal system were also used to assess the bonding quality based on the surface morphology and the presence of potential defects.

### 2.4. Bonding Strength Test Methods

**Figure 2:**
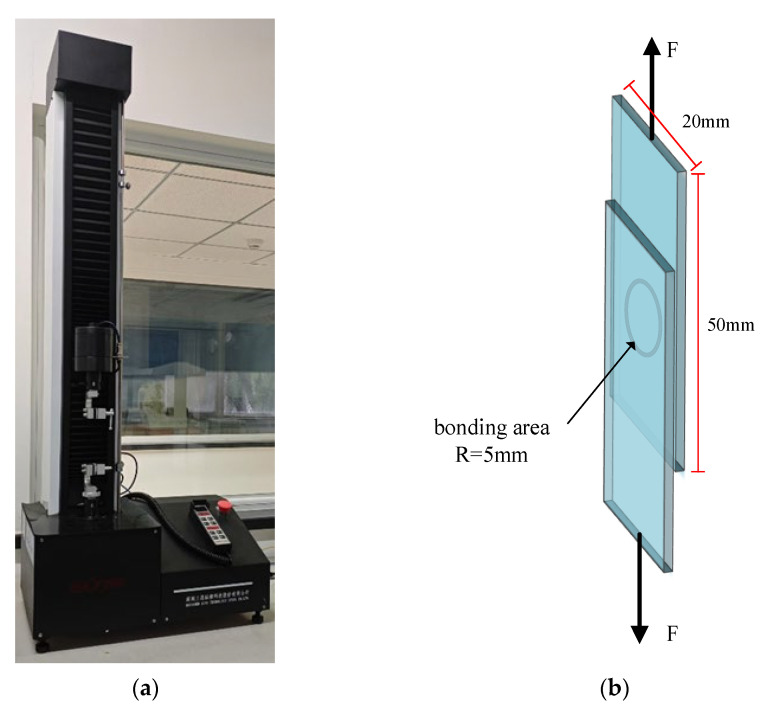
Schematic diagram of connection strength test equipment and test method. (**a**) Electronic universal testing machine, (**b**) Connection strength test diagram.

In this experiment, the electronic universal testing machine, UTM2503, was used to measure the connection strength of the glass sample after bonding. The connection strength is characterized by the shear strength of the sample. The geometry of the sample is 20 mm × 50 mm × 1.5 mm, and the radius of the bonding area is 5 mm. The test was conducted in quasi-static mode, with a loading speed of 1 mm/min and a data sampling interval of 0.002 s. The force gradually increases as the test progresses. The test method is shown in Figure 2b.

During the experiment, the tensile testing machine applied parallel and opposite forces to the material, recorded the maximum force *F*_max_ when the sample bonding weld broke, and calculated the connection strength according to the formula:

(2) $$\sigma =\frac{F_{\max}}{S}$$

In the equation, *S* is the area of the bonding weld.

### 2.5. Exploratory Experiments of Ultrafast Laser Bonding of Multilayer Glasses of Different Thicknesses

When the middle layer of the microfluidic chip is so thin that the depth of focus of the laser beam can cover the middle layer and part of the upper and lower layers of glass, we can choose to bond multiple layers of glass at once. The focus of the laser is set in the center of the laminated glass, glass and the laser interaction results in nonlinear absorption ionization, so that the glass can be bonded.

In laser processing systems, the depth of focus of the laser beam refers to the distance from the focal point when the light intensity at a point on the optical axis is reduced to half of the focal point of the beam.

(3) $$z=\pm \frac{\lambda f^{2}M^{2}}{\pi A^{2}r_{0}^{2}}$$

In the equation, the λ is the laser wavelength, the f is the focal length of the scanning galvanometer, the $M^{2}$ is the laser quality factor, the A is the magnification of the beam expander, and the $r_{0}$ is the diameter of the laser spot.

The depth of focus of the picosecond laser used in this experiment is calculated to be ±191.19 μm.

In this experiment, 20 mm × 50 mm × 1.5 mm soda-lime glass and 20 mm × 20 mm × 0.05 mm high alumina silicate glass were used, which was firstly cleaned with anhydrous ethanol for 5 min, then ultrasonically cleaned with deionized water for 5 min in order to wash away dust, stains, and impurities on the surface, and then finally dried and prepared for use.

**Figure 3:**
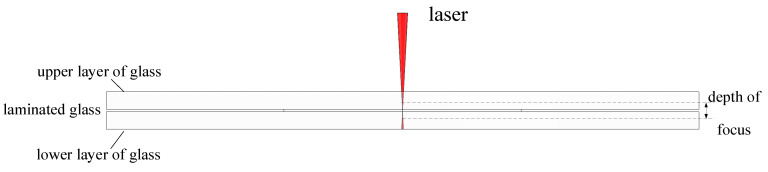
Schematic diagram of ultrathin laminated glass bonding.

After determining the focus position using the focusing lens and ITO glass, the clean and dry three pieces of glass were stacked in natural dislocation, as shown in Figure 3, to make a close fit, and the laser focus was moved to the center of the laminated glass by using Equation (1) to calculate the distance after deflection.

We proposed a method of bonding multilayer glass with variable focus for the laser bonding of arbitrary thickness laminated glass, which avoids the positioning errors caused by multiple positioning during the process, and greatly improves the processing accuracy and efficiency.

In this experiment, 20 mm × 50 mm × 1.5 mm soda-lime glass was used, which was firstly cleaned with anhydrous ethanol for 5 min, then ultrasonically cleaned with deionized water for 5 min in order to wash away dust, stains, and impurities on the surface, and then finally dried and prepared for use.

**Figure 4:**
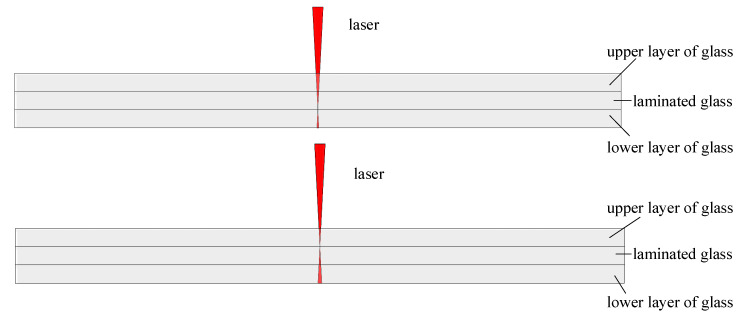
Schematic diagram of variable focus laser bonding of multilayered glass.

As shown in Figure 4, in the variable focus laser bonding of multilayer glass, the laser focus is first placed between the lower glass and the laminated glass to complete the bonding of them. And then the laser focus is shifted upward and placed between the upper glass and the laminated glass, and then scanned again to complete the bonding of the multilayer glass. The reason for adopting this processing sequence is to prevent the upper glass from modifying after laser scanning, which affects the laser absorption of the lower glass. Compared with the traditional bonding method using PDMS film or dispensing bonding, this method requires only one positioning, which greatly reduces the positioning error and improves processing efficiency. If the upper and lower flow paths are complex and do not overlap, this method can complete the bonding with only one positioning, which provides a new direction for the design and manufacture of microfluidic chips.

## 3. Results

### 3.1. Effect of Single Pulse Energy on Weld Morphologies

The laser output power (0–20 W) is controlled by adjusting the Modulated Control Setpoint (MCS) on the diode driver. The correspondence between the power code and output power is shown in Table 3.

At 1064 nm wavelength and 10 ps pulse width, the laser ablation threshold of soda-lime glass is about 9.54 J/cm^2^ [24].

The relationship between the single pulse energy and the average laser output power could be calculated by Equation (4):

(4) $$E_{P}=\frac{P_{\mathrm{avg}}}{f}$$

In the equation, the $P_{\mathrm{avg}}$ is the average laser output power, the f is the laser repetition frequency.

The single pulse energy affects the form of ionization within the glass and also has an effect on the type of ablation. Fix repetition frequency of 500 kHz, scanning speed of 1000 mm·s^−1^, 30 repetitions of the scan and defocusing amount of +80 μm. The energy of a single pulse is set as 14.1 μJ, 15.2 μJ, 16.5 μJ, 17.9 μJ, 19.6 μJ, 20.9 μJ, 22.0 μJ and 24.6 μJ. Observe the effect of single pulse energy on weld morphologies.

When the single-pulse energy is below 13.5 μJ, it fails to reach the ablation threshold. Multiphoton ionization is insufficient, avalanche ionization does not occur, and no molten-bond formation was observed. A single pulse energy of 14.1 μJ. At that time, the two glasses were not successfully bonded, but there were traces of laser ablation on the surfaces to be bonded. The reason is that the nonlinear absorption effect between the laser and the material is not strong enough, and although a melting phenomenon is produced, the melted glass is not enough to fill the gap between the bonded surfaces, leading to the failure of the bonding.

Weld width, heat-affected zone width, and weld morphology are critical parameters for evaluating the quality of laser welding. An appropriate weld width indicates a stable molten pool and sufficient bonding, contributing to high mechanical strength and good hermeticity. If the weld is too narrow, incomplete fusion may occur, while an overly wide weld can lead to ablation or collapse of the material. The heat-affected zone width reflects the extent of thermal diffusion—an excessively wide heat-affected zone usually implies excessive heat input, which can cause residual stress or thermal cracking, especially in thermally brittle materials such as glass. Weld morphology directly reflects the stability of the welding process. Ideally, the weld should be smooth and continuous, without defects such as splatter, pores, or cracks, all of which are crucial for long-term reliability. Therefore, optimizing laser power, scanning speed, and focusing conditions to achieve uniform weld width, narrow heat-affected zone, and high-quality surface morphology is essential for improving laser welding performance.

**Figure 5:**
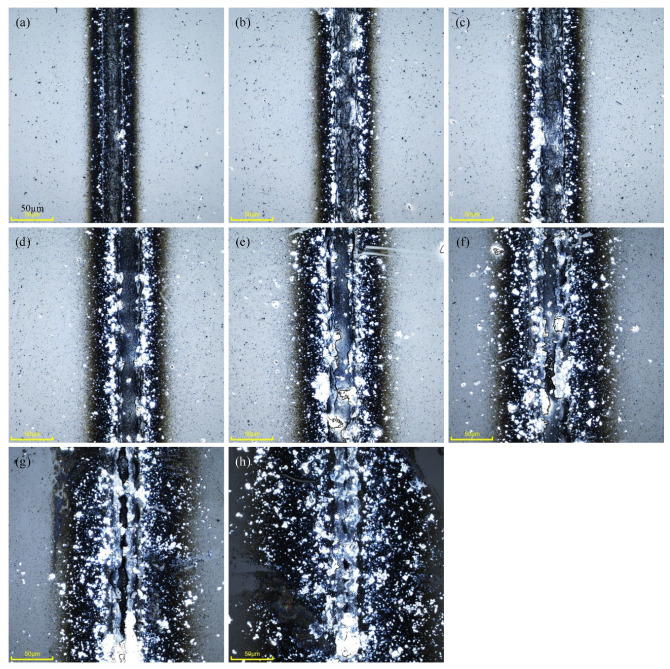
Morphologies of glass-bonded welds at different single pulse energies (scale bar: 50 μm), (**a**) 14.1 μJ, (**b**) 15.2 μJ, (**c**) 16.5 μJ, (**d**) 17.9 μJ, (**e**) 19.6 μJ, (**f**) 20.9 μJ, (**g**) 22.0 μJ and (**h**) 24.6 μJ.

As the single pulse energy continued to increase, as shown in Figure 5, the weld surrounding is discolored due to the heat influence, the range of the heat-affected zone increased, and the weld morphologies changed. For example, as shown in Figure 5b, at a single pulse energy of 15.2 μJ, the weld is smooth and neat, the heat-affected zone is small, and the range of molten spatter is small. With the increase of the single pulse energy, as shown in Figure 5d,e, the weld gradually develops an irregular shape, the heat-affected zone expands, and the presence of molten spatter increases. Finally, as shown in Figure 5h, the weld has developed a large number of notches and chipping, while the heat-affected zone and spatter range are at their maximum. With the single pulse energy increase, processing damage is produced, and a large amount of heat will be deposited in the laser action range, resulting in stress concentration; therefore, micro-cracks and other defects will be formed in the bonding region. Because the natural stacking state of the glass has a certain gap, molten glass in the laser-induced shock wave is generated by the role of the splash to the gap between the glass, and finally solidifies. When raising the single pulse energy, the shock wave energy will also be raised, and the melt will be able to splash to a more distant location.

**Figure 6:**
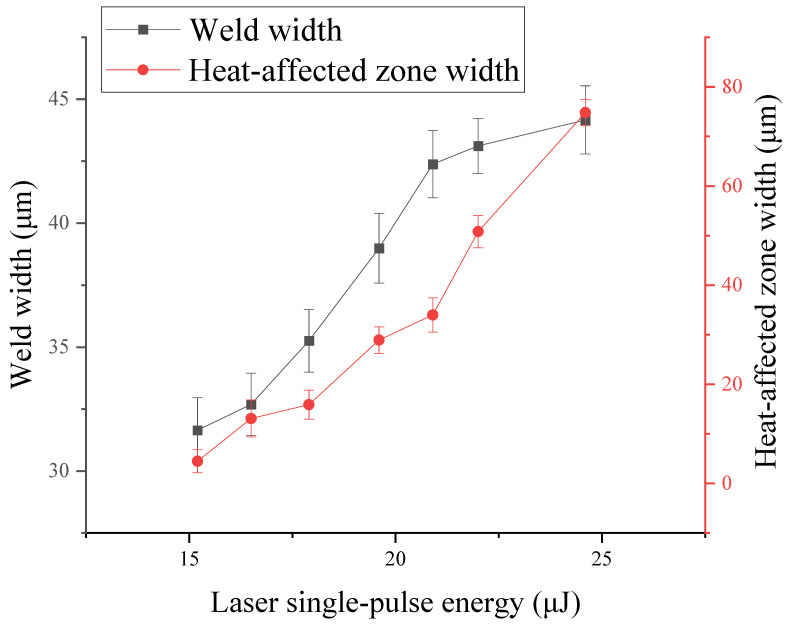
Relationship between laser single-pulse energy and weld width and heat-affected zone width.

In each group of experimental parameters of five experiments, three points at the weld seam are taken, and the average value of their weld width is taken as the width of the weld. Then, take the width of the heat-affected zone on the right side of the weld as the width of the heat-affected zone range. As shown in Figure 6, it can be seen that weld width increases moderately with single-pulse energy, rising from 31.65 μm at 14.1 μJ to 44.16 μm at 24.6 μJ. The extent of the heat-affected zone is strongly influenced by the energy of the single pulse, and at the single pulse energy of 15.2 μJ, the width of the heat-affected zone is 4.472 μm. When the energy of a single pulse is 24.6 μJ, the width of the heat-affected zone is 74.822 μm. In the case of single pulse energies exceeding 19.6 μJ, the expansion of the heat-affected zone increases. When increasing the laser single pulse energy to a higher level, the range of action will deposit a lot of energy, causing the heat-affected zone to continue to expand, and the deepening of the color represents that the microstructure has been changed. In conclusion, when selecting the single pulse energy, it is necessary to consider the bonding power, weld morphologies and heat-affected zone range and other factors. In order to avoid bonding failure at low energy and weld damage at high energy, the single pulse energy is selected as 16.5–19.6 μJ, where the favorable weld morphologies are achieved.

### 3.2. Effect of Laser Scanning Speed on Weld Morphologies

**Figure 7:**
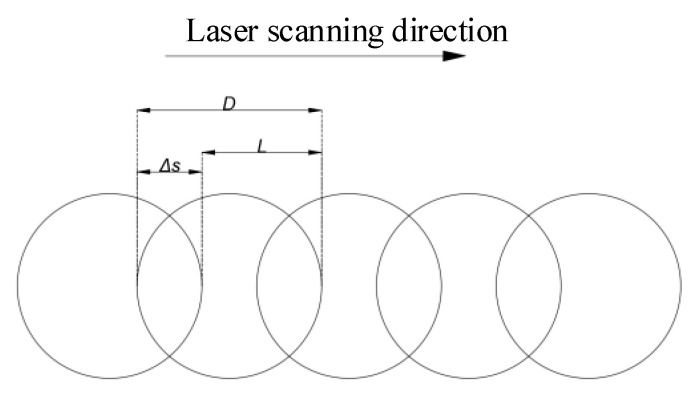
Schematic diagram of the overlap rate of the laser spot.

Laser scanning speed affects the laser spot overlap rate, which refers to the laser overlap area width and spot diameter ratio in the laser scanning direction, as shown in Figure 7.

Spot Overlap Ratio σ can be obtained from Equation (5):

(5) $$\sigma =\frac{\Delta s}{D}\times 100\%=(1-\frac{v}{Df})\times 100\%$$

In the equation, the f is the laser repetition frequency, the v is the laser scanning speed.

In this experiment, the movement of the galvanometer system was used instead of the conventional displacement platform, so that the scanning speed was improved by two to three orders of magnitude, which greatly improved the bonding efficiency. Setting the experimental process parameters: laser single pulse energy 19.6 μJ, scanning speed was gradually increased from 50 mm·s^−1^ to 3000 mm·s^−1^, while the laser repetition frequency was 500 kHz and the number of laser scans was kept at 30 times constantly.

**Figure 8:**
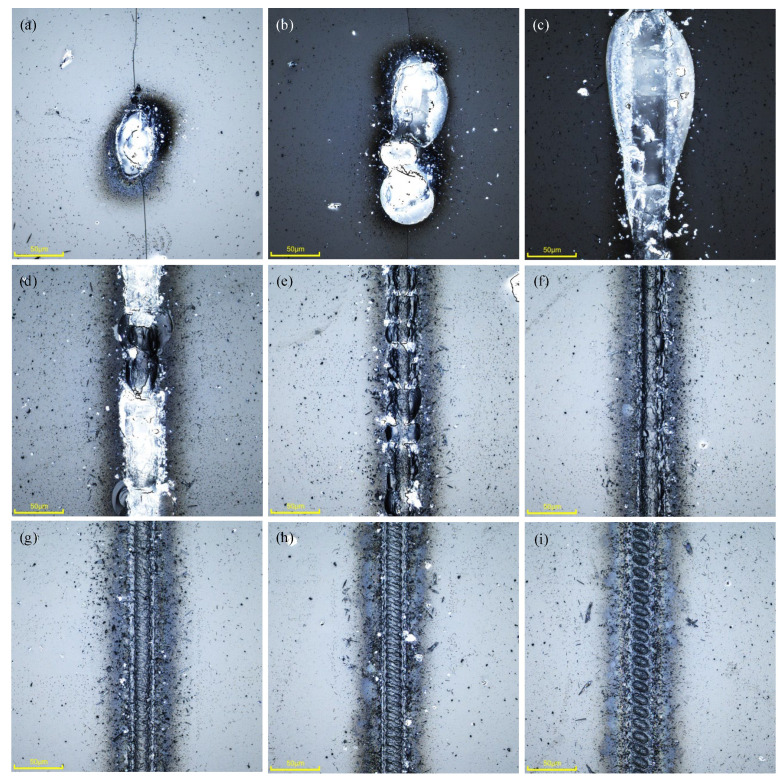
Morphologies of glass-bonded welds at different scanning speed (scale bar: 50 μm), (**a**) 100 mm·s^−1^, (**b**) 200 mm·s^−1^, (**c**) 400 mm·s^−1^, (**d**) 600 mm·s^−1^, (**e**) 800 mm·s^−1^, (**f**) 1000 mm·s^−1^, (**g**) 1500 mm·s^−1^, (**h**) 2000 mm·s^−1^ and (**i**) 3000 mm·s^−1^.

The relationship between different scanning speeds and the spot overlap rate is shown in Table 4. The relationship between the weld morphologies and the scanning speed is shown in Figure 8. The experimental results show that when the laser scanning speed was below 400 mm·s^−1^ (the spot overlap rate reaches 95%), the bonding area cannot form a continuous, uniform weld. When the scanning speed is 50 mm·s^−1^, cracks are generated and spread; When the speed is 100 mm·s^−1^, false welds appear at the weld, and the actual weld has been burned, as shown in Figure 8b. The reason is that too many effective laser pulses at the same position, amounting to 62.6, result in a large amount of plasma in the area of action, which produces micro-explosions and impedes the downward propagation of the laser light, and thus a uniform weld seam cannot be formed. When the scanning speed is 400 mm·s^−1^, a continuous weld can be formed between the glass, but there were chipping and defects, and the weld was rough, as shown in Figure 8c. This is because the higher number of laser pulses will make the temperature of the region rise faster, causing the heat of the glass expansion and extrusion with the surrounding material, resulting in chipping and defects. In addition, the inability to expel air from the weld area in a timely manner at low scanning speeds can also lead to the formation of defects such as porosity. As the scanning speed continues to increase, the edge of the weld gradually becomes smooth. And when the speed is 1500 mm·s^−1^, the straightness of the edge of the weld is good, as shown in Figure 8g. Continuing to increase the scanning speed at this point will result in micro-creases in the weld, while the weld morphologies become rough again. Too fast scanning speed does not allow the glass material to melt completely, resulting in a non-smooth joint with a semicircular boundary, as shown in Figure 8i. When the scanning speed is increased to 3500 mm·s^−1^, the glass will not be successfully bonded.

**Figure 9:**
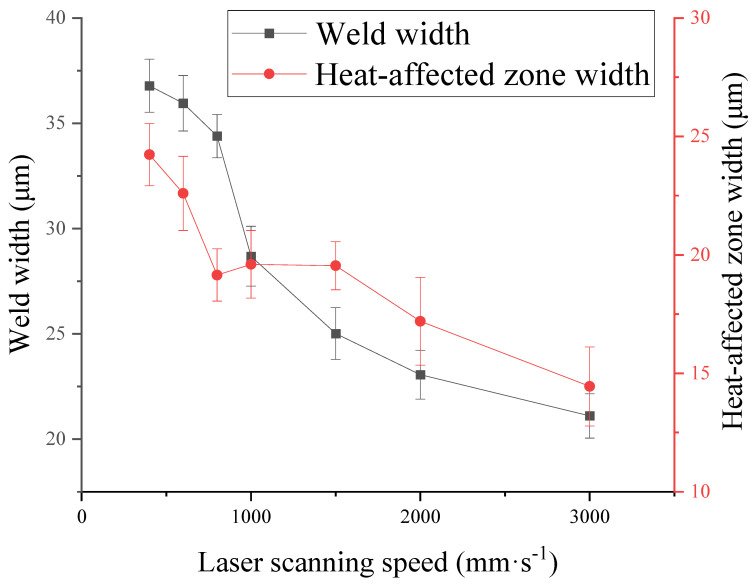
Relationship between laser scanning speed and weld width and heat-affected zone width.

Make statistics on weld width and heat-affected zone width at different scanning speeds, as shown in Figure 9. With the increase in laser scanning speed, the width of the weld and heat-affected zone decreases, because the accumulated laser energy will decrease. When the scanning speed is less than 400 mm·s^−1^, uniform welds cannot be formed, and when the scanning speed is faster than 3000 mm·s^−1^, both sides of the weld are rough. When selecting bonding process parameters, we should pay attention to the relationship between weld morphologies and scanning speed, and choose a moderate scanning speed.

### 3.3. Effect of Laser Repetition Frequency on Weld Morphologies

Laser repetition frequency refers to the number of laser pulses per unit time, which affects the weld morphologies by affecting the energy accumulation in the material. The higher the repetition frequency, the stronger the energy accumulation effect. Set the laser pulse energy to 19.6 μJ, the laser spot overlap rate is 90%, the scanning times are 30 times, the laser scanning speed is 1000 mm·s^−1^ constantly, and the laser repetition frequency is set to 300 kHz, 400 kHz, 500 kHz, 550 kHz, 600 kHz and 650 kHz, respectively. Observe the influence of laser repetition frequency on weld morphologies.

**Figure 10:**
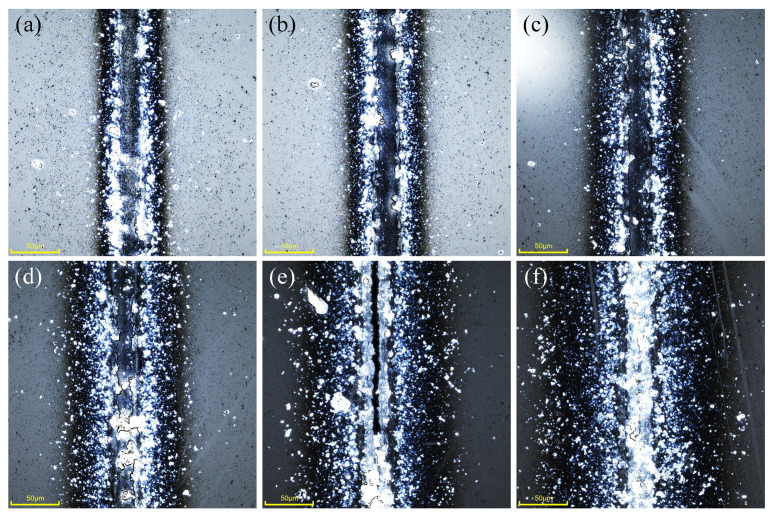
Morphologies of glass-bonded welds at different repetition frequency (scale bar: 50 μm), (**a**) 300 kHz, (**b**) 400 kHz, (**c**) 500 kHz, (**d**) 550 kHz, (**e**) 600 kHz and (**f**) 650 kHz.

The relationship between different laser repetition frequencies and weld morphologies is shown in Figure 10. As can be seen from the figure, with the increase of laser repetition frequency, the defects at the weld gradually increase. When the repetition frequency is 300 kHz, the spattering distance of the melt is shorter. Because the laser frequency is smaller and the interval between laser pulses is longer. Therefore, before the arrival of the next laser pulse, the material has a longer time to transfer energy, which is not conducive to the accumulation of energy and the melting of the material, so the temperature of the material is lower and the spattering distance of the melt is shorter. With the increase of laser repetition frequency, the cracks and defects of the weld will increase, and the increase of repetition frequency will also accelerate the accumulation of energy until the material melts to complete bonding.

**Figure 11:**
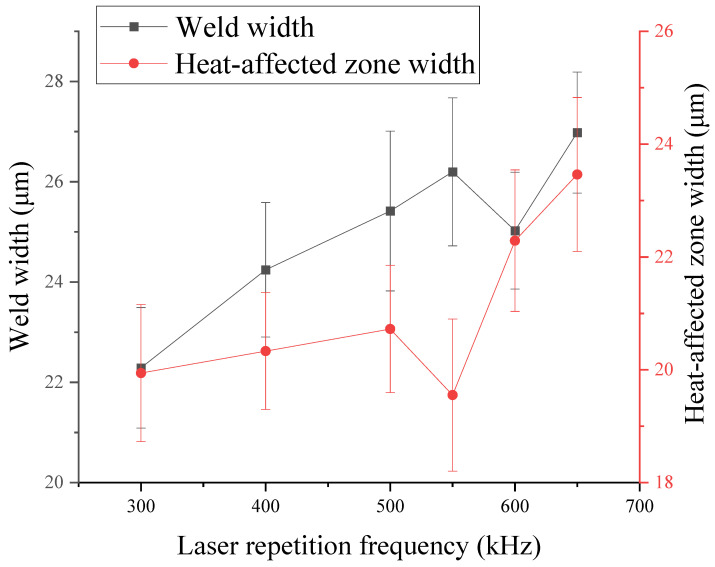
Relationship between laser repetition frequency and weld width and heat-affected zone width.

The relationship between laser repetition frequency and weld width and heat-affected zone width is shown in Figure 11. When the laser repetition frequency is 300 kHz, the weld width and the heat-affected zone width are the smallest. With the increase of the laser repetition frequency, the weld width and the heat affected zone width increase, and they both reach the maximum when the laser repetition frequency is 650 kHz. Especially, when the laser repetition frequency is above 500 kHz, the range of the affected zone is accelerated, which may be caused by the stronger thermal accumulation effect. Therefore, in order to improve the thermal accumulation effect and bonding effect, it is best to choose a higher laser repetition frequency.

### 3.4. Influence of Laser Scanning Times on Weld Morphologies

The laser scanning times refer to the number of times the laser scans repeatedly on the same machining path, which mainly affects the number of effective laser pulses. Set the laser pulse energy to 19.6 μJ, the laser spot overlap rate is 90%, the laser scanning speed is 1000 mm·s^−1^, the laser repetition frequency is 500 kHz, and the laser scanning times are set to 5, 10, 15, 30, 45, 60 and 90 times respectively. The relationship between scanning times and weld morphologies is explored.

**Figure 12:**
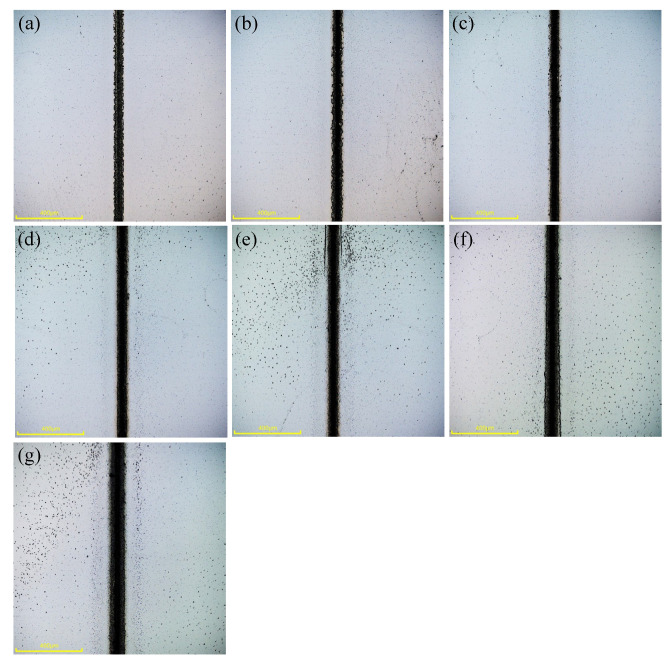
Morphologies of glass-bonded welds at different laser scanning times (scale bar: 50 μm), (**a**) 5 times, (**b**) 10 times, (**c**) 15 times, (**d**) 30 times, (**e**) 45 times, (**f**) 60 times and (**g**) 90 times.

The relationship between the laser scanning times and weld morphologies is shown in Figure 12. As shown in Figure 12a, when the scanning time is 5, the heat-affected zone is very small and there is basically no molten spatter. Weld width is narrow, the surface is rough, and there are notches, chipping and other defects on both sides of the weld. Longitudinal cracks exist above the weld parallel to the direction of laser incidence, this is because the number of scans is too small, the effective number of pulses is small, the volume of the material to be melted is small, so the width of the weld is narrow, the molten material receives a small laser-induced pressure, basically will not be spattered. In addition, in the cooling process of the glass, the melted glass is drawn by the weld on both sides of the glass, resulting in cracks. As shown in Figure 12b,c, when the scanning times are 15 and 30 times, the quality of the weld is better and the straightness is higher. With the increase in the number of laser scanning times, the heat-affected zone around the weld is gradually expanded, and the range of molten material spatter is farther away. When the number of scanning times is larger than 45 times, the quality of the weld will reduce, and chipping and defects will appear. This is because, as the number of scanning times increases, the effective pulses increase at the same position of the material, the glass material accumulates too much energy, and the excessively melted glass material may produce uneven shrinkage and cracks during cooling. In addition, multiple scans increase the exposure time of the weld to air, increasing the possibility of oxidation reactions, which can affect the quality of the weld and introduce impurities that can lead to the formation of micro-cracks. Therefore, too many laser scanning times will lead to a reduction in the quality of the weld.

**Figure 13:**
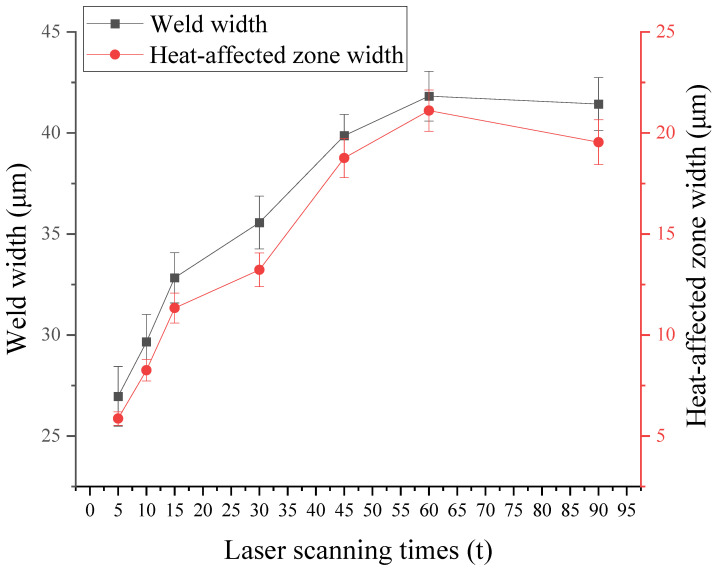
Relationship between laser scanning times and weld width and heat-affected zone width.

The relationship between laser scanning times and weld width and heat-affected zone width is shown in Figure 13. Weld width and heat-affected zone width show an increasing trend with the increase of the scanning times, the width increases faster when the scanning times are 5~45 times, and the width is basically unchanged when the scanning times are more than 45 times, which indicating that the scanning times have a small influence on the weld width and heat-affected zone width. When selecting process parameters, it is recommended to choose moderate parameters to avoid defects such as micro-cracks brought by low scanning times and the degradation of weld morphologies quality brought by too high scanning times.

### 3.5. Ultrafast Laser Bonding Connection Strength Test Experiment

**Figure 14:**
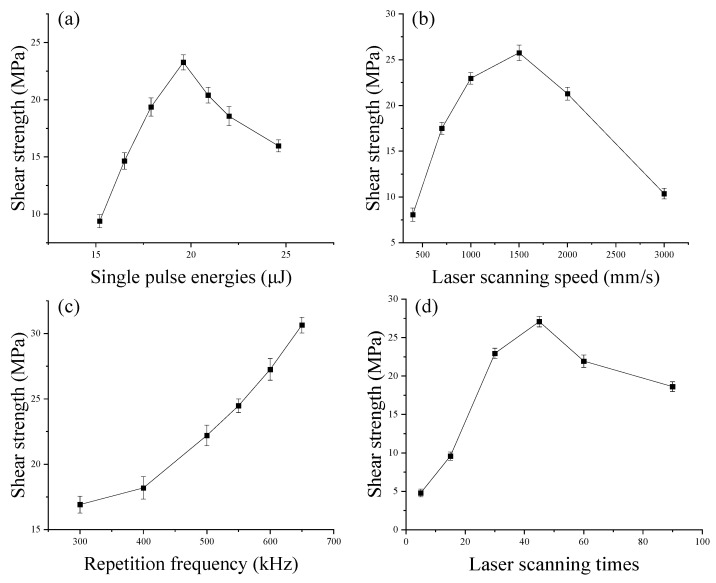
Effect of process parameters on shear strength.

It can be seen from Figure 14a that the shear strength increases first and then decreases with the increase of single pulse energy. When the single pulse energy increases from 15.2 μJ to 19.6 μJ, the shear strength increases linearly; when the single pulse energy decreases from 19.6 μJ to 24.6 μJ, the shear strength also decreases. At the same time, the downward trend slows down in the range of single pulse energy from 20.9 μJ to 24.6 μJ. When the laser single pulse energy is 19.6 μJ, the shear strength of the sample reaches a maximum value of 23.272 MPa. When the laser single pulse energy is 15.2 μJ, the energy density can be obtained to be 9.93 J/cm^2^, which is slightly higher than the single pulse ablation threshold of soda-lime glass. At this time, the material absorbs laser energy and ionization occurs inside, but it cannot generate enough seed electrons through the nonlinear absorption effect. At this time, the material can absorb laser melting, but the molten material forms a small molten pool, the width of the weld is relatively small, and the bonding strength is also small, which is the minimum value of 9.38 MPa. As the energy of a single laser pulse increases, the nonlinear absorption effect generated by the interaction between the laser and the material gradually increases, the seed electrons generated by multiphoton ionization increase, and then avalanche ionization is triggered, which modifies the material, improves the energy absorption rate of the material in the modified area, expands the molten pool, and increases the connection strength between materials. When the single pulse energy is greater than 19.6 μJ, due to the excessive increase in energy, ablation latent heat is easily formed in the laser action area, and defects in the bonding weld gradually increase, which has an adverse effect on the connection strength, so the shear strength shows a decreasing trend. Therefore, 19.6 μJ is selected during bonding to obtain a bonding weld with a beautiful structure and high strength.

Figure 14b shows the change trend of the shear strength of the glass-bonded sample at different laser scanning speeds. As the laser scanning speed continues to increase, the shear strength of the sample tends to rise first and then fall. When the scanning speed is 400 mm/s, the minimum shear strength of the sample is 8.06 MPa. When the laser scanning speed is in the range of 400–1500 mm/s, the shear strength of the sample increases from 8.06 MPa to 25.75 MPa. From the scanning speed of 1500 mm/s, the shear strength of the sample begins to decrease. When the scanning speed is 3000 mm/s, the shear strength of the sample is 10.36 MPa. When the laser scanning speed is 400 mm/s, the overlap rate of the laser is high, reaching 94%. At this time, the number of effective laser pulses at the same position after one scan reaches 15.7. At this time, too many effective pulses per unit area lead to excessive local thermal stress. Combined with the experimental results in Section 3.2, when the scanning speed is too slow, cracks, edge collapse and other defects will form around the bonding weld, resulting in low shear strength. When the scanning speed is too fast, the spot overlap rate is low. At this time, due to the small number of effective laser pulses at the same position, the temperature rise is limited, the molten pool area is small, and the gap-filling degree between materials is limited, resulting in a decrease in the shear strength of the sample. At the same time, when the laser scanning speed is fast, micro-wrinkles appear in the bonded weld and semicircular notches appear on both sides, and longitudinal cracks appear on the weld surface. These defects will also lead to a decrease in shear strength. Therefore, when optimizing the process, a moderate scanning speed (1000~1500 mm·s^−1^) should be selected to avoid the formation of defects.

It can be seen from Figure 14c that as the laser repetition frequency increases, the shear strength of the sample also increases. When the repetition frequency is 300 KHz, the minimum shear strength of the sample is 16.19 MPa. When the laser repetition frequency is in the range of 300–400 KHz, the shear strength increases slowly, from 16.19 MPa to 18.18 MPa, while when the laser frequency is 400–650 MPa, the shear strength of the sample increases at a faster rate, from 18.18 MPa to 30.64 MPa. Under the conditions of constant scanning speed and laser single pulse energy, the higher the repetition frequency, the more obvious the heat accumulation effect, the greater the amount of material melting and the greater the bonding strength. Combined with the experimental results in Section 3.3, when the laser repetition frequency increases, the defects of the material increase slightly but are less obvious. At the same time, when the laser repetition frequency is greater than 500 KHz, the width of the bonded weld also increases rapidly, which also confirms the effective improvement of the bonding effect by heat accumulation. It can be concluded that it is more appropriate to use a 600 kHz or 650 kHz for laser bonding.

Figure 14d shows the trend of the shear strength of the glass-bonded sample under different laser scanning times. As the number of laser scans increases, the shear strength of the glass shows a trend of first increasing and then decreasing. When the scanning times are 5, the minimum shear strength of the sample is 4.79 MPa, and when the number of scans is 45, the maximum shear strength of the sample is 27.08 MPa. When the scanning times are within the range of 5–30, the shear strength of the sample increases rapidly. This is because at the beginning of the laser action, the glass material is in a cold state and gradually heats up under the action of the laser. When the scanning times are low, the glass material is only preheated, and the volume of the molten pool formed is not enough to produce enough molten material to fill the gaps between the glasses. As the scanning times increase, the material temperature increases significantly, the volume of the molten pool expands, and the material undergoes reversible or irreversible thermal expansion, making the connection between the samples stronger. However, as the scanning times continue to increase, cracks and other defects gradually appear around the bonding weld. At the same time, as the thermal pressure induced by the laser gradually increases, the shock wave formed will cause the quality of the bonding weld morphology to decrease, and the bonding strength of the sample will gradually decrease. Therefore, considering the shear strength and processing efficiency comprehensively, in actual processing, the scanning times should be 45.

## 4. Exploratory Experiments of Ultrafast Laser Bonding of Multilayer Glasses of Different Thicknesses

Multilayer microfluidic chips have important applications in medical and biological fields, such as the detection of cancer [25]. Multilayer microfluidic chips can improve the detection efficiency. The bonding of multilayer glass is important in the manufacturing process of microfluidic chips, but the traditional bonding method requires several positioning steps during the bonding, which affects the accuracy and efficiency. In this section, we categorized and investigated the laser bonding of laminated glass with different thicknesses, and provided the methods of laser bonding of laminated glass with different thicknesses.

### 4.1. Exploratory Experiments of Laser Bonding of Ultra-Thin Laminated Glass

During the welding process, multiple small circles along the outer circular path were first scanned by spot welding, aiming at a shorter air gap distance and a uniform fit. The inner circular region was scanned by concentric circular paths with fixed spacing, so that the glass material and the laser continuously formed a nonlinear absorption effect and increased heat accumulation through multiple scans. This method creates a laterally developing melt pool at the interface of glass, and the increased melt can fill the gap between the glass better.

**Figure 15:**
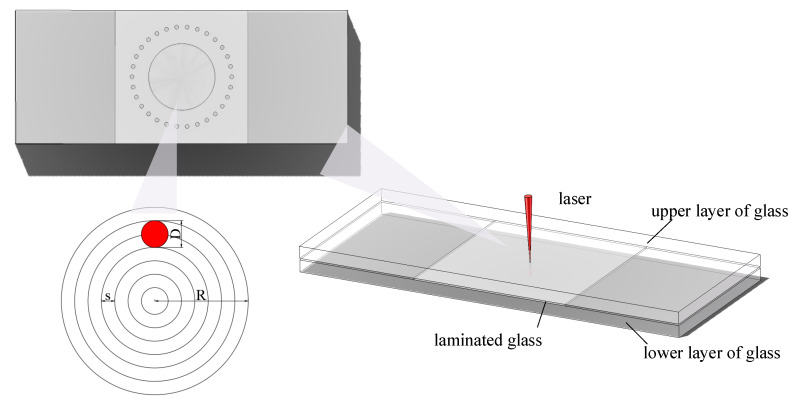
Schematic diagram of the bonding method.

As shown in Figure 15, the process parameters of laser bonding are divided into two parts. The first part is the spot welding of the outer ring, the radius of the circular path is 7.5 mm, the radius of each small circle is 0.1 mm, the single pulse energy is 19.6 μJ, the repetition frequency is 500 kHz, the scanning speed is 600 mm·s^−1^, and the scanning times are 150. The second part is the internal concentric circle path, the spacing of the rings denoted by s is 10 μm, the laser spot diameter denoted by *D* is 20 μm, the total radius of the concentric rings denoted by R is 5 mm, the single pulse energy is 16.5 μJ, the repetition frequency was 500 kHz, the scanning speed is 1000 mm·s^−1,^ and the scanning time is 60.

**Figure 16:**
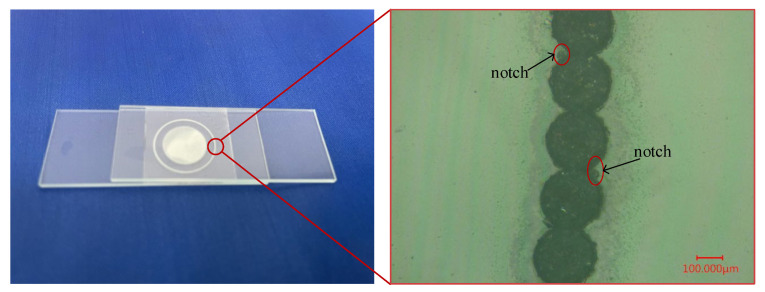
Physical picture and partial enlargement of multilayer glass bonding.

Figure 16 is a picture of the glass after bonding. The white opaque part of the picture is the bonding region, and stable interference fringes can be observed around it, indicating that the spacing between the materials has been reduced and the bonding has been successful.

Using a microscope to observe the bonding area of the outer ring, it can form a continuous bonding weld due to the dense weld joints, the heat-affected zone appears on both sides, and the molten spatters can be observed. The shape of the spot weld area is regular, the weld joints have good roundness, and there are more defects at the junction of the weld joints because the repeated scanning of the laser at the junction generates more plasma, which leads to local micro-explosions and produces notches and chipped edges, and the sharp corners on the structure also lead to more prone to stress concentration phenomena, which aggravate the generation of defects.

### 4.2. Exploratory Experiments of Variable Focus Laser Bonding of Multilayered Glass

In the above section, the laser bonding of ultrathin laminated glass was investigated, which is applicable to the case where the thickness of the interlayer is below 100 μm.

**Figure 17:**
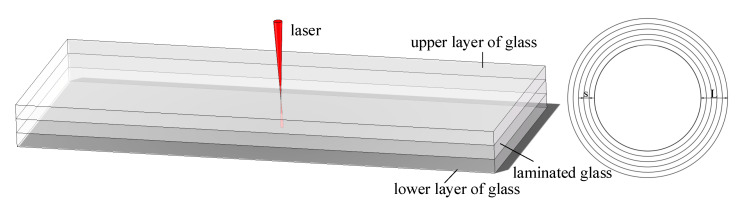
Schematic diagram of the bonding path.

Based on Section 3.1 (the effect of process parameters on weld morphologies), we set four different groups of process parameters, as shown in Table 5. Each set of parameters was repeated three times, and then the results were obtained. In the fourth group, a two-step scanning method was used, in which the sample was firstly scanned as a laser with a larger single pulse energy and a smaller repetition frequency, and then scanned as a laser with a smaller single pulse energy and a higher repetition frequency. As shown in Figure 17, the bonding paths use concentric rings with a ring spacing denoted by s is 5 μm and the width denoted by L of the circle is 0.5 mm.

**Figure 18:**
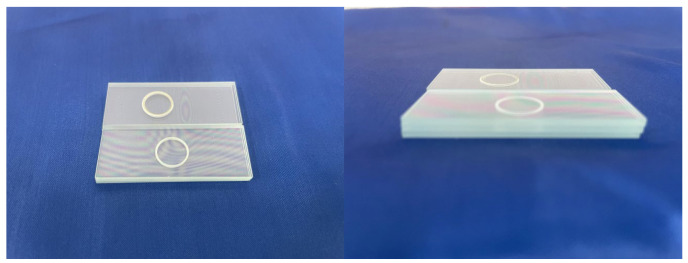
Physical picture of variable focus laser bonding of multilayered glass.

The physical picture of variable focus laser bonding of multilayered glass is shown in Figure 18. In the figure, the bonding welds of samples No. 1–3 are white, while the bonding weld color of sample No. 4 shows a slight yellowish color. In fact, sample No. 4 shows a burnt yellow color after the first scan, which is due to the photodarkening phenomenon that occurs in the glass material after a large single pulse radiation. In addition, the higher single pulse energy produces a small number of color centers and micro-void defects in the material near the contact surface, which can promote the absorption of laser energy during the next scan [26].

**Figure 19:**
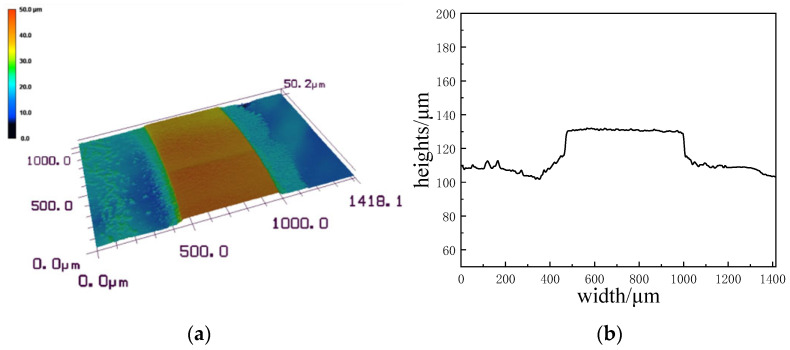
Localized weld morphologies. (**a**) Three-dimensional topography, (**b**) Profile drawing of the weld.

The three-dimensional topography of the weld seam was observed by a laser confocal scanning microscope, and the results are shown in Figure 19. The weld in the figure is uniform and regular, and we can see that the molten spatters on both sides of the weld and burst to a far distance, which is caused by the high scanning times. The actual width of the weld is 525.053 μm. The spot radius of picosecond lasers is 10 μm. The theoretical width of the weld is 520 μm. The actual width corresponds to the theoretical width, so the spherical aberration caused by focusing the laser in soda-lime glass is negligible.

## 5. Conclusions

We first examined ultrafast-laser bonding parameters for soda-lime glass, analyzing how single-pulse energy, scan speed, repetition frequency, and scanning times affect weld morphology and connection strength. Optimal parameter ranges were identified: single pulse energy is 19.6 μJ, scanning speed is 1000~1500 mm·s^−1^, laser repetition frequency is 600 kHz or 650 kHz, and the scanning time is 45. These optimized parameters resulted in a maximum shear strength of 30 MPa, demonstrating the potential for high-performance applications. Next, we conducted exploratory experiments on multilayer glass bonding. For ultra-thin interlayers (<100 µm), we demonstrated single-step bonding; for thicker stacks, we introduced a variable-focus approach. Both methods were experimentally validated. After the experiments, both methods can successfully achieve the purpose, avoid repetitive positioning, improve the bonding accuracy and processing efficiency.

## Figures and Tables

**Table 1:** Detailed parameters of the picosecond laser.

Performance	Parameters
Wavelength (nm)	1064
Maximum output power (w)	20
Repetition frequency (kHz)	100–1000
Pulse width (ps)	10
Beam quality	M2 ≤ 1.3
Output spot diameter (μm)	20
Typical scan angle (rad)	±0.35
Tracking error (ms)	0.14
Marking speed (rad·s^−1^)	2.5
Positioning speed (rad·s^−1^)	12.0
Scanning speed (mm·s^−1^)	0–4000
Spot Roundness	≥80%
Cooling method	Water-cooled
Preheating time (min)	10

**Table 2:** Physical properties of soda-lime glass.

Physical Quantities	Values
Thermal conductivity (W·m^−1^·K^−1^)	0.93
Density (g·cm^−3^)	2.5
Specific heat capacity at constant pressure (J·g^−1^·K^−1^)	1.10
Strain points (°C)	500
Softening point (°C)	730
Refractive index	1.52
Transmittance	90% (1.5 mm)
Expansion coefficient (K^−1^)	9 × 10^−6^ (20~300 °C)

**Table 3:** The correspondence between the MCS and the average output power.

MCS	200	250	300	350	400	600	800	1000
Average laser output power (W)	0.88	1.56	2.43	3.37	4.64	8.97	11.8	12.33
laser fluence values (J/cm^2^)	5.60	9.93	15.47	21.45	29.53	57.09	75.10	78.47

**Table 4:** The correspondence between the laser scanning speed and the spot overlap rate.

Scanning Speed (mm·s^−1^)	Spot Overlap Rate
50	99.5%
100	99%
200	98%
400	96%
600	94%
800	92%
1000	90%
1500	85%
2000	80%
3000	70%

**Table 5:** Experimental parameters of variable focus laser bonding of multilayered glass.

No	Repetition Frequency (kHz)	Single-Pulse Energy (μJ)	Scanning Speed (mm·s^−1^)	Scanning Times
1	500	19.6	1000	45
2	500	19.6	1500	45
3	500	16.5	1500	45
4	200/1000	44.8/8.97	600/50	10/10

## Data Availability

The authors confirm that the data supporting the findings of this study are available within the article.
